# Supplementary material for: Anterior and posterior retrosplenial cortex form distinct visuospatial circuits in the mouse
Source: Nat Commun. 2026 Mar 25;17:4388. doi: 10.1038/s41467-026-70762-z (PMC13181125; doi:10.1038/s41467-026-70762-z)
Supplement: Supplementary file 1 — Supplementary Information [file 41467_2026_70762_MOESM1_ESM.pdf]

SUPPLEMENTARY INFORMATION

1 Supplementary Tables

Supplementary Table 1: Summary of animals used for *in vivo* two-photon calcium imaging.

MOUSE	AGE*1 (weeks)	SEX	HEMI-SPHERE	FOV*2 AM (mm)	FOV*2 PM (mm)	FOV*2 AL (mm)	FOV*2 PL (mm)	GENOTYPE*3	NUMBER OF SESSIONS *4
**YW006**	20	M	Left	**A:** 0, -1.04<br>**P**: -0.23, -2.74	**A:** 0, -2.17<br>**P**: -0.23, -3.74	**A:** -0.67, -1.17<br>**P**: -0.9, -2.74	**A:** -0.67, -2.17<br>**P**: -0.9, -3.74	Thy1	**A**: 2 (depths)<br>**P**: 2 (depths)
**YW008**	20	M	Left	**A:** 0, -1.13<br>**P**: -0.28, -3.17	**A:** 0, -2.13<br>**P**: -0.28, -4.17	**A:** -0.67, -1.13<br>**P**: -0.9, -2.74	**A:** -0.67, -2.13<br>**P**: -0.95, -4.17	Thy1	**A**: 2 (depths)<br>**P**: 2 (depths)
**YW010**	22	M	Left	**A:** 0, -1.22<br>**P**: -0.12, -2.33	**A:** 0, -2.22<br>**P**: -0.12, -3.33	**A:** -0.67, -1.22<br>**P**: -0.79, -2.33	**A:** -0.67, -2.22<br>**P**: -0.79, -3.33	Thy1	**A**: 1<br>**P**: 1
**YW017**	22	M	Left	**A:** -0.22, -1.21<br>**P**: -0.2, -2.32	**A:** -0.22, -2.21<br>**P**: -0.2, -3.32	**A:** -0.89, -1.21<br>**P**: -0.87, -2.32	**A:** -0.89, -2.21<br>**P**: -0.87, -3.32	Thy1	**A**: 1<br>**P**: 1
**YW018**	22	M	Left	**A:** 0, -1.27<br>**P:** -0.2, -3.05	**A:** 0, -2.32<br>**P:** -0.2, -4.07	**A:** -0.65, -1.27<br>**P:** -0.88, -3.05	**A:** -0.65, -2.32<br>**P:** -0.88, -4.07	Thy1	**A**: 1<br>**P**: 1
**YW022**	25	M	Left	**A:** -0.2, -1.02<br>**P**: 0, -2.61	**A:** -0.2, -2.02<br>**P**: 0, -3.61	**A:** -0.87, -1.02<br>**P**: -0.67, -2.61	**A:** -0.87, -2.02<br>**P**: -0.67, -3.61	CaMKII	**A**: 1 (ETL)<br>**P**: 1 (ETL)
**YW032**	20	M	Left	**A:** 0, -1.2<br>**P**: 0, -2.94	**A:** 0, -2.38<br>**P**: 0, -3.94	**A:** -0.67, -1.38<br>**P**: -0.67, -2.94	**A:** -0.67, -2.38<br>**P**: -0.67, -3.94	CaMKII	**A**: 1 (ETL)<br>**P**: 1 (ETL)
**YW033**	20	M	Left	**A:** 0, -1.2<br>**P**: -0.1, -2.68	**A:** 0, -2.2<br>**P**: -0.1, -3.68	**A:** -0.67, -1.2<br>**P**: -0.77, -2.68	**A:** -0.67, -2.2<br>**P**: -0.77, -3.68	CaMKII	**A**: 1 (ETL)<br>**P**: 1 (ETL)
**YW036**	20	M	Left	**A:** -0.2, -1.49<br>**P:** -0.2, -3	**A:** -0.04, -2.48<br>**P:** -0.2, -4.1	**A:** -0.86, -1.59<br>**P:** -0.9, -3.02	**A:** -0.71, -2.59<br>**P:** -0.9, -4.1	CaMKII	**A**: 1 (ETL)<br>**P**: 1 (ETL)
**YW045**	20	M	Left	**A:** -0.15, -0.88<br>**P**: 0, -1.87	**A:** -0.15, -1.95<br>**P**: 0.15, -3.01	**A:** -0.87, -0.88<br>**P**: -0.78, -1.97	**A:** -0.87, -1.95<br>**P**: -0.6, -3.15	CaMKII	**A**: 1<br>**P**: 1 (ETL)
**YW048**	20	M	Left	**A:** -0.05, -1.038<br>**P**: -0.13, -2.59	**A:** 0, -2.14<br>**P**: 0, -3.67	**A:** -0.78, -1.09<br>**P**: -0.83, -2.69	**A:** -0.66, -2.19<br>**P**: -0.71, -3.76	CaMKII	**A**: 1 (ETL)<br>**P**: 1 (ETL)
**YW049**	20	M	Left	**A:** -0.19, -0.64<br>**P**: -0.16, -2.64	**A:** 0, -1.56<br>**P**: -0.03, -3.67	**A:** -0.96, -0.64<br>**P**: -0.83, -2.71	**A:** -0.8, -1.45<br>**P**: -0.72, -3.75	CaMKII	**A**: 1 (ETL)<br>**P**: 1 (ETL)
**YW071**	40	M	Left	**A:** 0, -0.83<br>**P:** -0.21, -2.74	**A:** 0, -1.74<br>**P:** -0.21, -3.65	**A:** -0.52, -0.83<br>**P:** -0.89, -2.74	**A:** -0.52, -1.74<br>**P:** -0.89, -3.65	CaMKII	**A:** 1 (ETL)<br>**P**: 1 (ETL)
**YW102**	20	F	Left	**A:** -0.27, -0.65<br>**P:** 0, -1.91	**A:** 0, -1.7<br>**P:** 0.18, -3.02	**A:** -1.06, -0.87<br>**P:** -0.8, -2.1	**A:** -0.8, -1.91<br>**P:** 0.64, -3.35	Thy1	**A:** 1 (ETL)<br>**P:** 1 (ETL)
**YW109**	20	F	Left	**A:** -0.1, -0.77<br>**P:** -0.1, -2.16	**A:** -0.1,-1.85<br>**P:** -0.1,-3.31	**A:** -0.94 -0.77<br>**P:** -0.94, -2.16	**A:** -0.94, -1.85<br>**P:** -0.94, -3.31	Thy1	**A:** 1 (ETL)<br>**P:** 1 (ETL)

***1.** Age for the imaging data collection

***2.** Field of view anchor coordinates: Imaging regions were anchored at specific coordinates relative to Bregma in anteromedial (AM), posteromedial (PM), anterolateral (AL), and posterolateral (PL) locations. The anterior RSC (A) and posterior RSC (P) were analyzed, with negative values indicating positions in the left hemisphere (x) or posterior relative to Bregma (y).

***3.** Thy1: Thy1-GCaMP6s; CaMKII: CaMKII-tTA x TRE-GCamp6.lineG6s2

***4.** Two animals (YW006 and YW008) were each recorded in two separate sessions on different days, with each session targeting a distinct imaging depth. ETL indicates that recordings were performed across four optical planes using an electrically tunable lens.

Supplementary Table 2: Summary of *in vivo* calcium imaging data.

MOUSE	Retinotopy	No tactile	Tactile	Tactile + darkness	Virtual reality
YW006	+	+	+		
YW008	+		+		
YW010	+	+	+		
YW017	+	+	+		
YW018		+			
YW022	+		+	+	
YW032	+	+	+	+	
YW033	+	+	+	+	
YW036		+		+	
YW045				+	
YW048	+	+	+	+	
YW049		+		+	
YW071					+
YW102					+
YW109					+

Supplementary Table 3: Summary of viral vector injections for retrograde tracing

MOUSE_ID/STRAIN / SEX	INJECTION AGE (weeks)	COORDINATES (mm) (AP/DV/ML)	VIRAL CONTRUCTS	SLICE PLANE
**YW043 / WT / male**	15	anterior: –1.5/1/0.4<br>posterior: –3.2/0.8/0.4	anterior: pAAV-hSyn-EGFP<br>posterior: pAAV-hSyn-mCherry	Coronal
**YW051 / WT / male**	17	anterior: –1.5/1/0.4<br>posterior: –3.2/0.8/0.4	anterior: pAAV-CAG-GFP<br>posterior: pAAV-CAG-tdTomato	Coronal
**YW063 / WT / male**	14	anterior: –1.5/1/0.4<br>posterior: –3.2/0.8/0.4	anterior: pAAV-CAG-tdTomato<br>posterior: pAAV-CAG-GFP	Coronal
**YW064 / WT / male**	14	anterior: –1.5/1/0.4<br>posterior: –3.2/0.8/0.4	anterior: pAAV-CAG-tdTomato<br>posterior: pAAV-CAG-GFP	Coronal

Supplementary Table 4: Key resources table

RESOURCE	SOURCE	IDENTIFIER
**Mouse line**		
Wild type	KU Leuven	RRID:IMSR_JAX:000664
Thy-1 GCaMP6 line	Jackson Laboratories	RRID:IMSR_JAX:024275
CaMKII GCaMP6 line	Jackson Laboratories	RRID:IMSR_JAX:024742; RRID:IMSR_JAX:007004
**Chemical and virus**		
pAAV-CAG-GFP	addgene	# 37825-AAVrg
pAAV-CAG-tdTomato	addgene	# 59462-AAVrg
pAAV-hSyn-EGFP	addgene	# 50465-AAVrg
pAAV-hSyn-mCherry	addgene	# 114472-AAVrg
Cholera Toxin Subunit B	ThermoFisher	# C34775
Vetergesic	Ceva Santé Animale	CNK:2623-627
Cefazolin	Sandoz	CNK:2218-204
Ketamine	Dechra	CNK:3120-060
Xylazine	VMD	CNK:1339001
Phosphate-buffered saline	Invitrogen	# AM9625
Histofix	Carl Roth	# P087.5
Agarose	Sigma	# A9793-100G
Mounting medium with DAPI	VECTASHIELD	# H-2000
UV Curing Optical Adhesives	Thorlabs	# NOA61
**Software and algorithms**		
Fiji	NIH	RRID:SCR_002285
MATLAB R2017-R2020	Mathworks	https://www.mathworks.com/
SHARP-Track	Shamash et al., 2018	https://github.com/cortex-lab/allenCCF
neuralib	Open Source	https://github.com/ytsimon2004/neuralib2
rscvp	Open Source	https://github.com/ytsimon2004/rscvp
posdc	Open Source	https://github.com/ytsimon2004/posdc
brainrender	Claudi et al., 2021	https://github.com/brainglobe/brainrender
suite2p	https://doi.org/10.1101/061507	https://github.com/mouseland/suite2p

2 Supplementary Figures

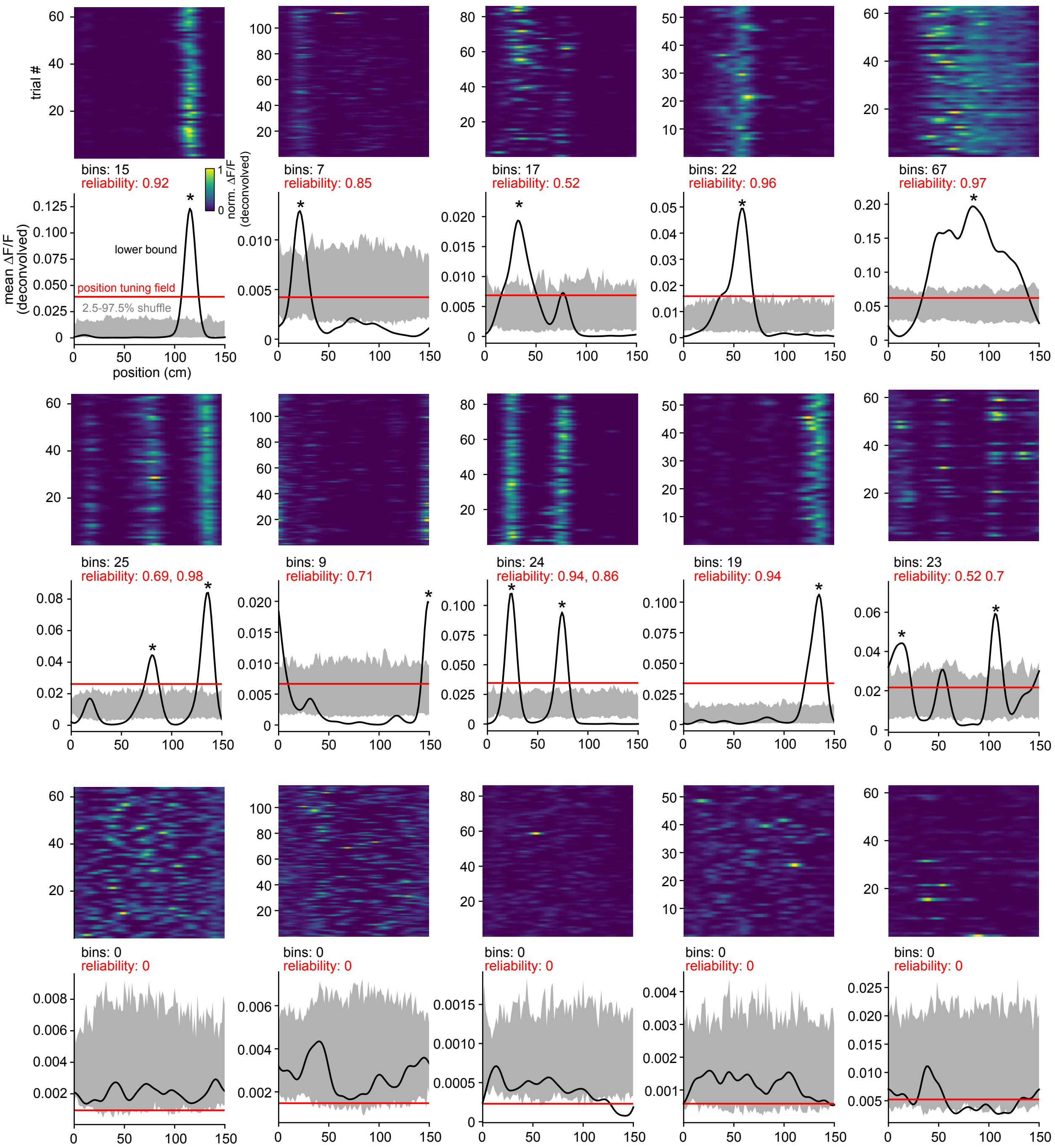

Supplementary Figure 1. Selection of position-tuned neurons

Position-tuned neurons were identified based on the reliability of their position-related responses and further refined using quantification of the position tuning field properties. The heatmaps show normalized, deconvolved $\Delta F/F_0$ signals from example RSC neurons, aligned to the animal's position across repeated laps. Neurons were classified as position-tuned if the lower bounds of their activity (mean – SEM) (black

lines) exceeded the 97.5th percentile of the shuffled distribution (grey shaded area) in at least one spatial bin. In addition, neurons were required to have a position tuning field width between 15 cm and 120 cm (red line), and to exhibit reliable activation, defined as the presence of a detectable position tuning field in more than one-third of trials (reliability > 0.33). Neurons meeting all criteria are marked with asterisks (*).

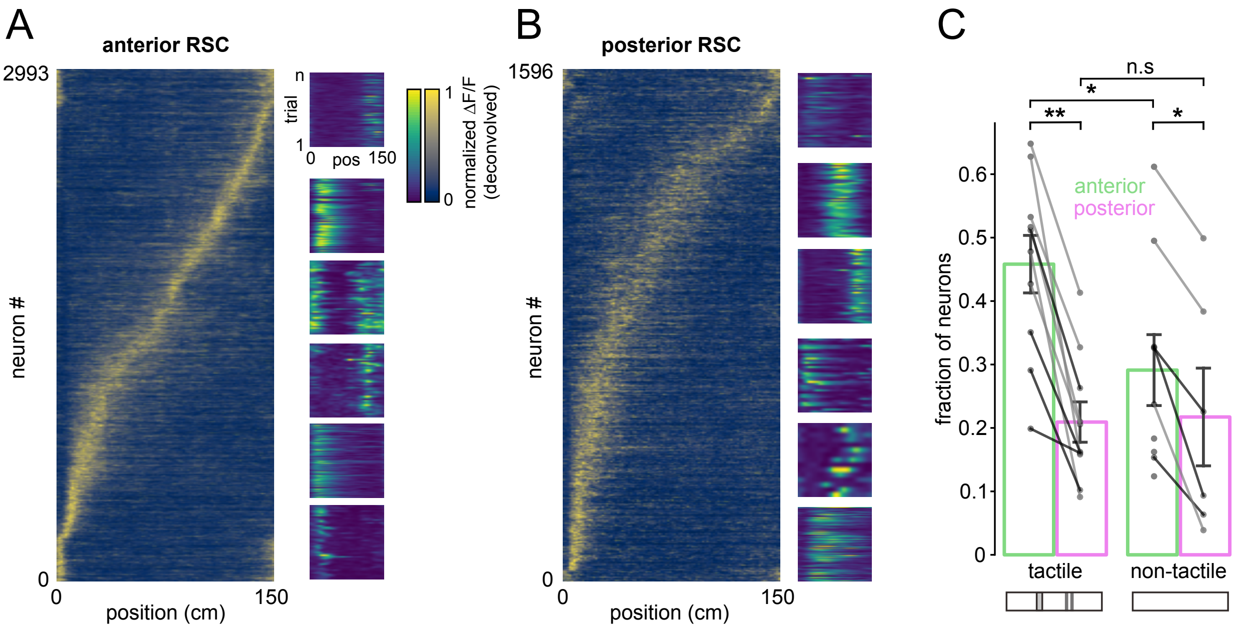

Supplementary Figure 2. Anterior–posterior gradient in position tuning is preserved in the absence of tactile cues

(A–B) Left: Cross-validated, trial-averaged deconvolved $\Delta F/F_0$ activity for all position-tuned neurons in RSC in the absence of tactile cues. Neurons are sorted by peak activity using one half of the trials and plotted using the other half. Right: Examples of normalized deconvolved $\Delta F/F_0$ signals from individual position-tuned neurons.

(C) Proportion of position-tuned neurons in anterior versus posterior RSC in tactile (same as Fig. 2A) and non-tactile treadmill conditions. Vertical bars indicate mean ± SEM across sessions; each connected line represents an individual animal (n = 10 sessions per group from 8 animals in tactile environment; n = 9 sessions from 9 animals in anterior RSC in non-tactile environment; n = 6 sessions from 6 animals in posterior RSC in non-tactile environment). Two-sided Mann–Whitney U test: anterior vs. posterior RSC, p = 0.002 (tactile), p = 0.03 (non-tactile); anterior RSC, tactile vs. non-tactile, p = 0.03; posterior RSC, tactile vs. non-tactile, p = 0.79.

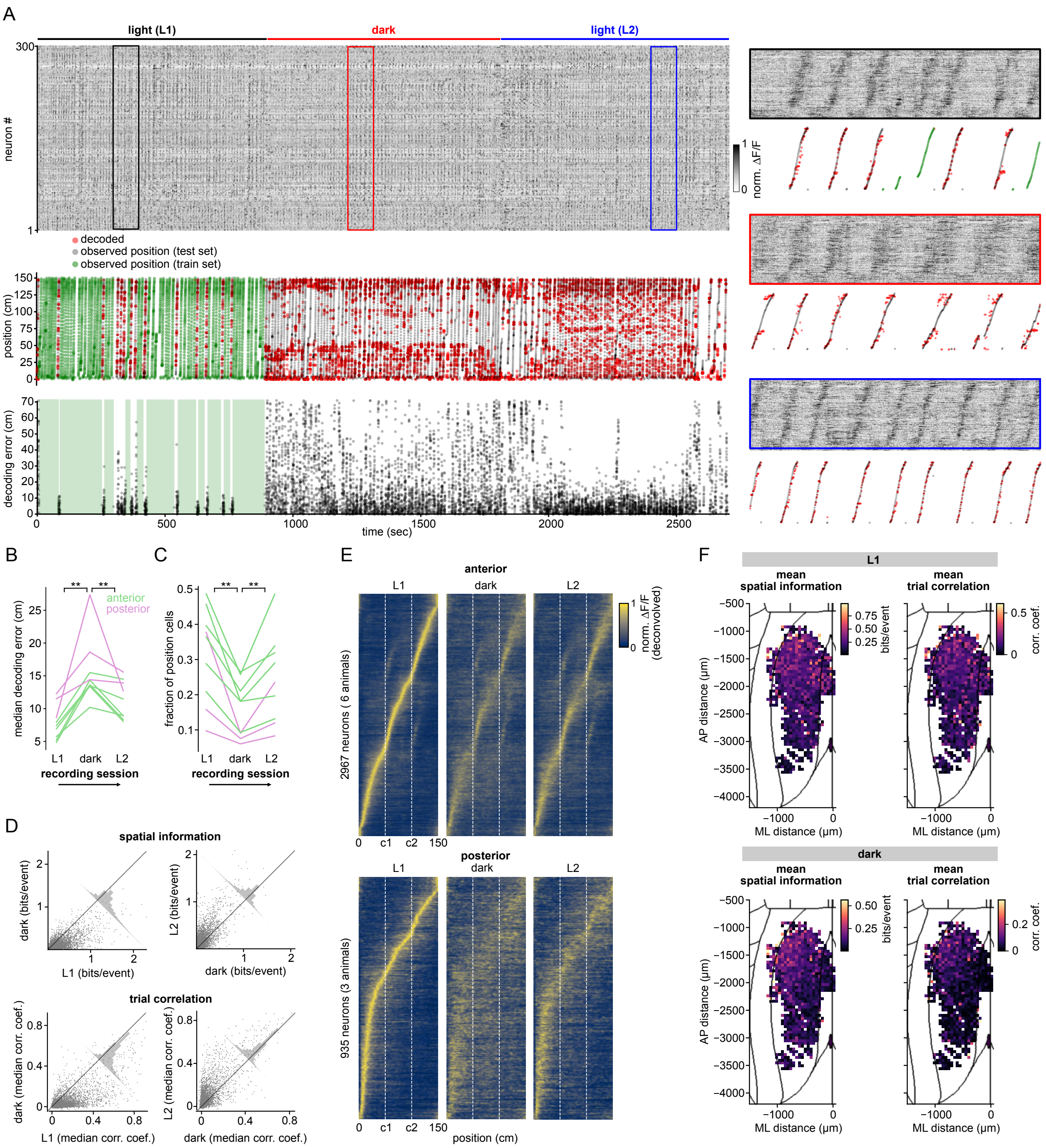

Supplementary Figure 3. RSC position coding in darkness

(A) Top row: Example population activity from 300 randomly selected neurons, with magnified segments displayed on the right. Second row: a Bayesian decoder trained on 80% of trials from the initial gray screen session (green) and evaluated on the remaining trials using 5-fold cross-validation. Observed positions from test trials are indicated by gray dots, while decoded positions are represented by red dots. Bottom row: Frame-by-frame decoding error, with trained trials indicated by green shading.

(B) Median decoding error across sessions. Two-sided Wilcoxon signed-rank test; light (L1) vs. dark; $p = 0.0039$; dark vs. light (L2); $p = 0.0039$ ($n = 9$ sessions from 7 animals).
(C) Fraction of position-tuned neurons across sessions. Two-sided Wilcoxon signed-rank test; light (L1) vs. dark; $p = 0.0039$; dark vs. light (L2); $p = 0.0039$ ($n = 9$ sessions from 7 animals).
(D) Pairwise scatter plots of spatial information and median trial-to-trial correlation across recording sessions, with histograms along the diagonal indicating the fraction of cells (3903 cells $n = 9$ sessions from 7 animals).
(E) Trial-averaged, deconvolved $\Delta F/F_0$ for all position-tuned neurons in anterior and posterior RSC across recording sessions, sorted according to activity from the initial gray screen session.
(F) Dorsal cortical map showing mean spatial information and mean trial-to-trial correlation across the RSC. Median values were binned every 50 μm and represented using a color scale ($n = 9$ sessions from 7 animals).

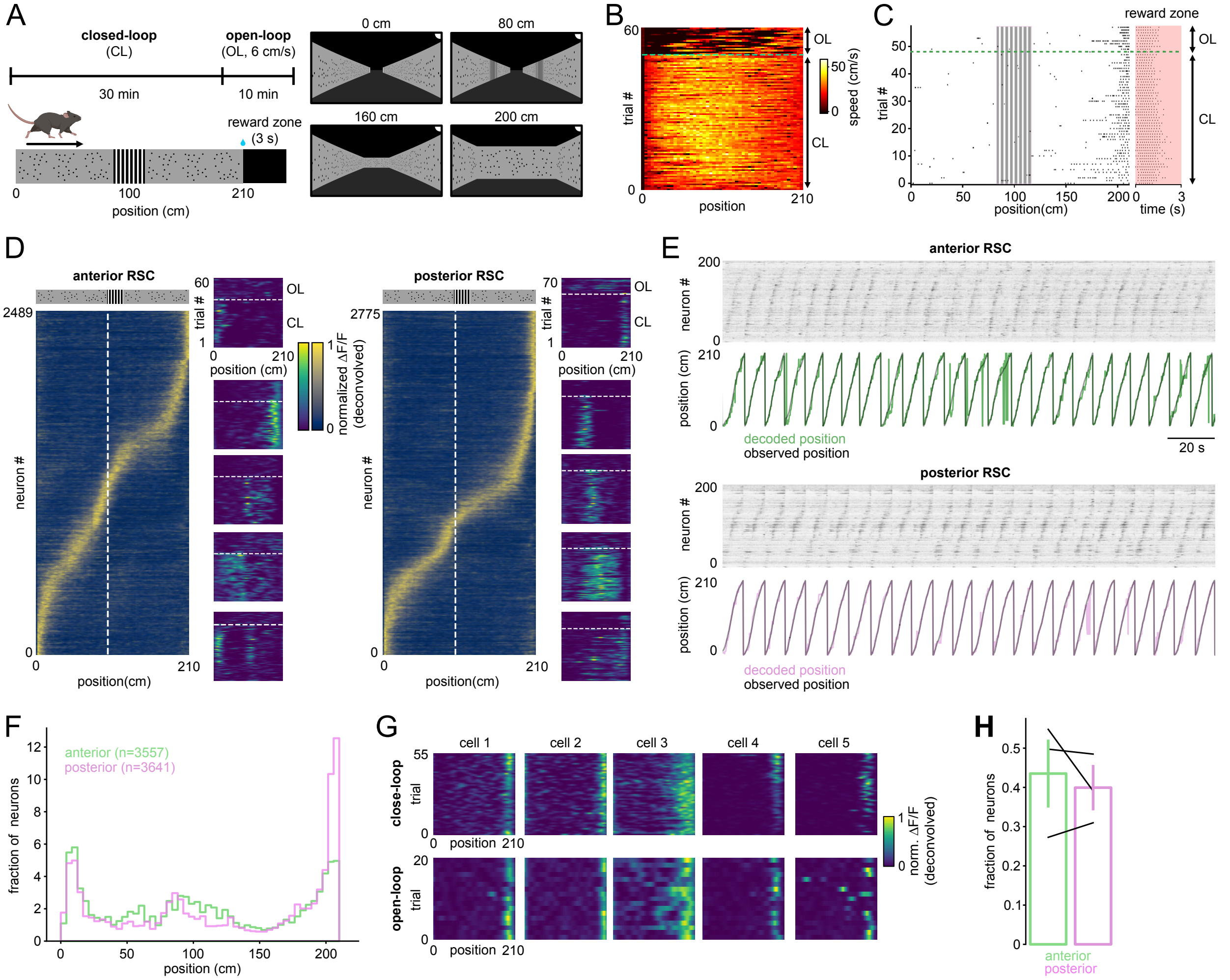

Supplementary Figure 4. RSC position responses in a virtual environment

(A) Schematic of the virtual reality task. Each session consisted of a 30-minute closed-loop condition (in which locomotion was coupled to visual scene movement), followed by a 10-minute open-loop condition (visual scene moved passively at a constant speed of 6 cm/s). A vertical grating landmark was placed at 100 cm along the virtual corridor. Mouse cartoon created in BioRender. Wei, Y. (2026) https://BioRender.com/c0ric0k.

(B) Heatmap showing running speed from a trained animal as a function of position across trials for a representative session.

(C) Example session showing a trained animal exhibiting anticipatory licking behavior before the reward zone.

(D) Trial-averaged deconvolved $\Delta F/F_0$ activity for all position-tuned RSC neurons (n = 3 sessions per group from 3 animals; anterior: 2,489 neurons, posterior: 2,775 neurons). Insets show examples of normalized $\Delta F/F_0$ traces from individual position-tuned neurons. Neurons are sorted by peak activity from one half of the trials and plotted using the other half (cross-validated).

(E) Top: Normalized calcium activity ($\Delta F/F_0$) from 200 randomly selected RSC neurons, sorted by the position of peak activity. Bottom: Decoded (green and magenta) and actual (black) positions across time from the same session.

(F) Histogram showing the distribution of position field peaks along the virtual corridor. Each neuron could contribute multiple position fields, resulting in 3,557 fields from 2,389 neurons (anterior RSC) and 3,641 fields from 2,775 neurons (posterior RSC).

(G) Five example neurons showing consistent responses to the end of the virtual corridor during both closed-loop and open-loop conditions.

(H) Proportion of position-tuned neurons in anterior vs. posterior RSC. Bars show mean ± SEM across sessions. Neurons with peak activity beyond 200 cm were excluded from quantification.

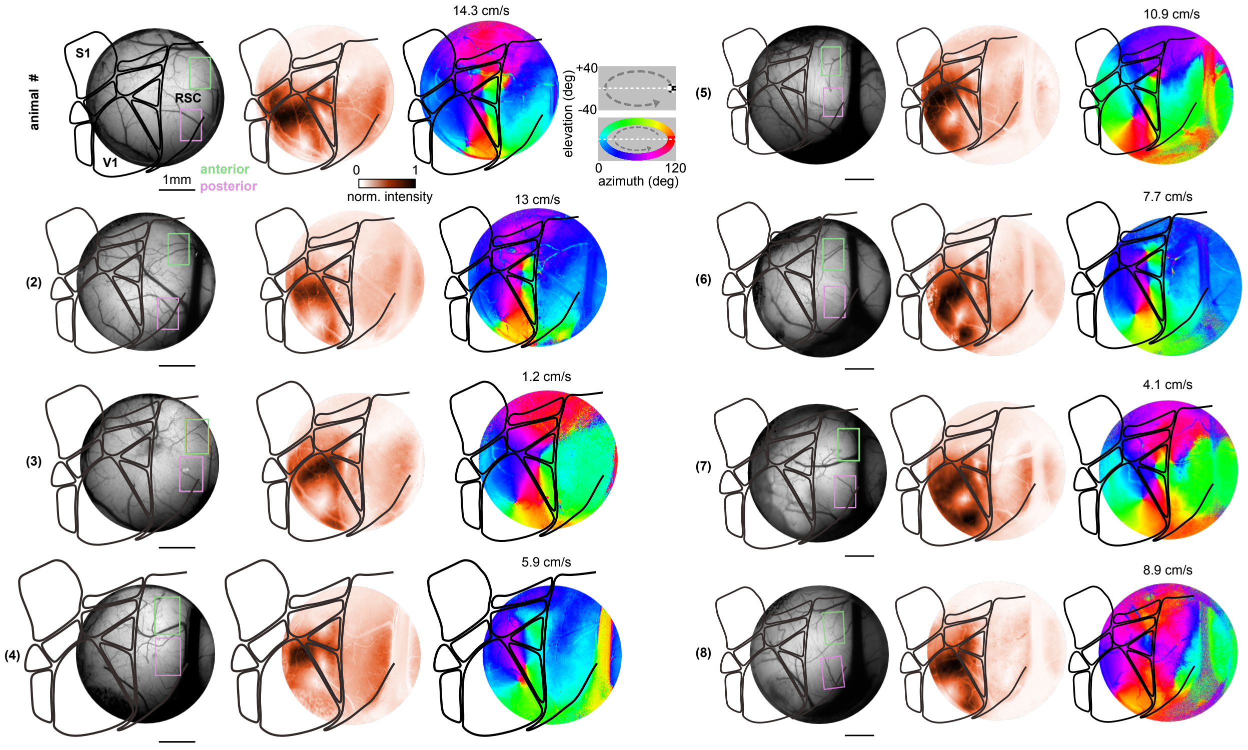

Supplementary Figure 5. Selection of RSC imaging fields of view based on visual retinotopy

Imaging fields of view (FOVs) were selected from animals listed in Supplementary Table 2. Left: bright-field image of the cranial window with outlines of inferred visual area borders (black) and selected FOVs (green, magenta). Middle: widefield calcium response amplitudes evoked by a rotating circular patch stimulus, color-coded by magnitude. Right: corresponding phase maps, where color indicates the preferred retinotopic location. The animal's running speed is shown at the top. Scale bar, 1 mm.

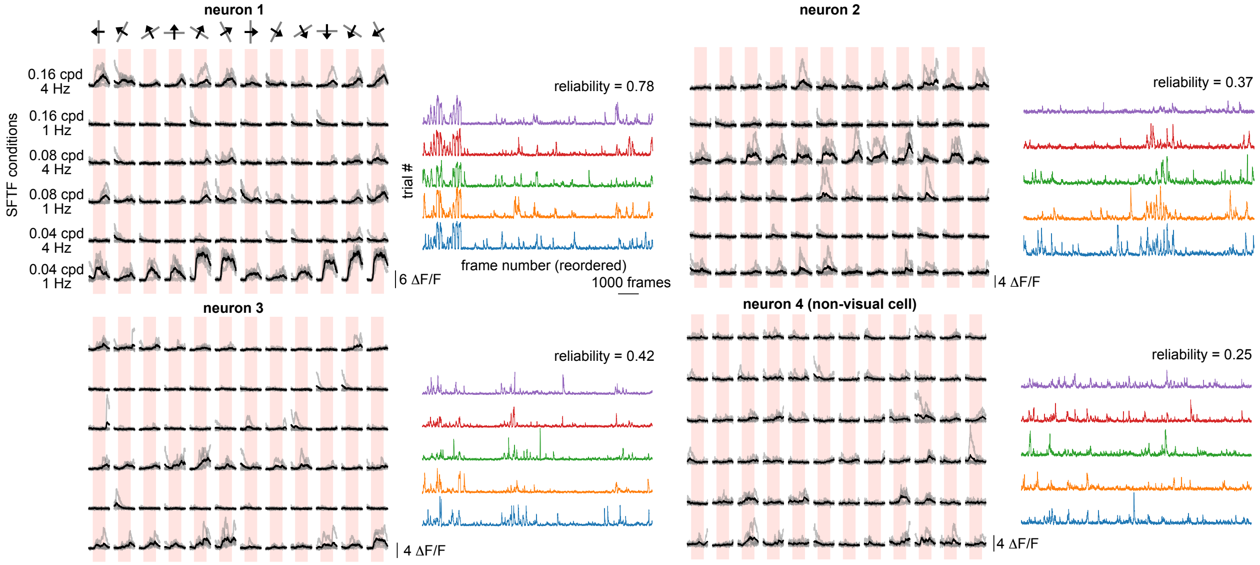

Supplementary Figure 6. Selection of visually responsive cells

Four example RSC neurons responding to drifting grating visual stimuli. The left panel shows responses across different combinations of spatial frequencies (0.04, 0.08, and 0.16 cpd) and temporal frequencies (1 and 4 Hz), with rows representing frequency conditions and columns indicating stimulus direction. The right panel presents the corresponding calcium signals ($\Delta F/F_0$), de-randomized by visual stimulus conditions during stimulation epochs, with colors denoting individual trials. Reliability index (r), computed as the 75th percentile of trial-to-trial Pearson correlation coefficients, is indicated above each panel.

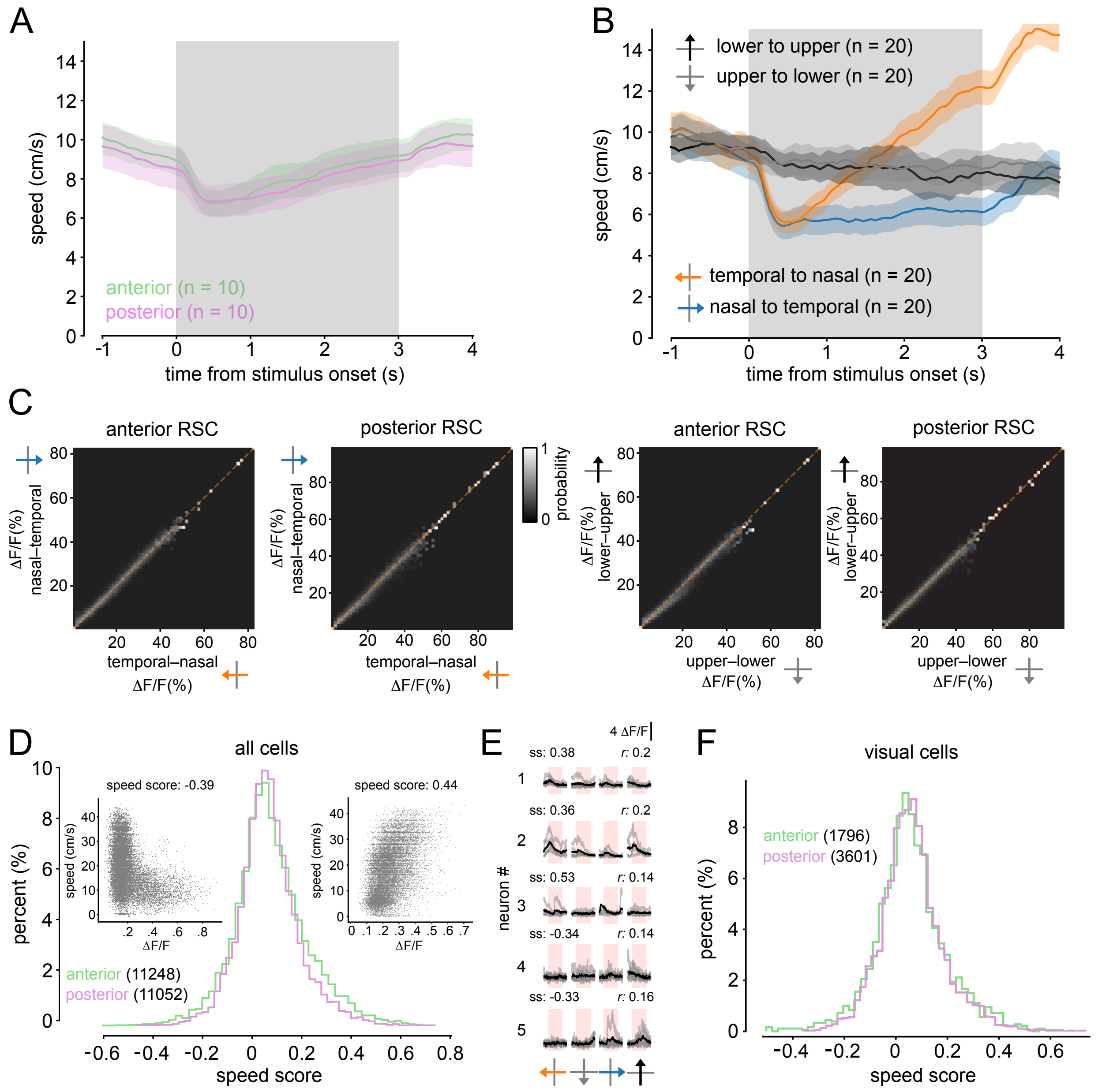

Supplementary Figure 7. Visual motion modulates running behavior, but speed and visual tuning are encoded by distinct neuronal populations in RSC

(A) Average running speed aligned to visual stimulus onset, shown separately for anterior and posterior RSC recordings (n = 10 sessions per group from 8 animals).

(B) Average running speed aligned to the onset of different visual motion directions, pooled across all recordings (n = 20 sessions).

(C) Maximum $\Delta F/F_0$ during the 3-second visual stimulation window for opposing motion directions: nasal-to-temporal vs. temporal-to-nasal, and upper-to-lower vs. lower-to-upper. Analyzed from 11,248 (anterior) and 11,052 (posterior) neurons (n = 10 sessions per group from 8 animals).

(D) Distribution of speed scores for all recorded neurons in anterior versus posterior RSC. Insets show

two example neurons showing negative and positive correlations between $\Delta F/F_0$ and running speed. 11,248 (anterior) vs. 11,052 (posterior) neurons (n = 10 sessions per group from 8 animals).

(E) Visual tuning curves for five example speed-correlated cells. Speed score (ss) and visual reliability (*r*) are shown above each trace.

(F) Distribution of speed scores for visually responsive neurons in anterior and posterior RSC. 1,796 (anterior) vs. 3,601 (posterior) neurons (n = 10 sessions per group from 8 animals).

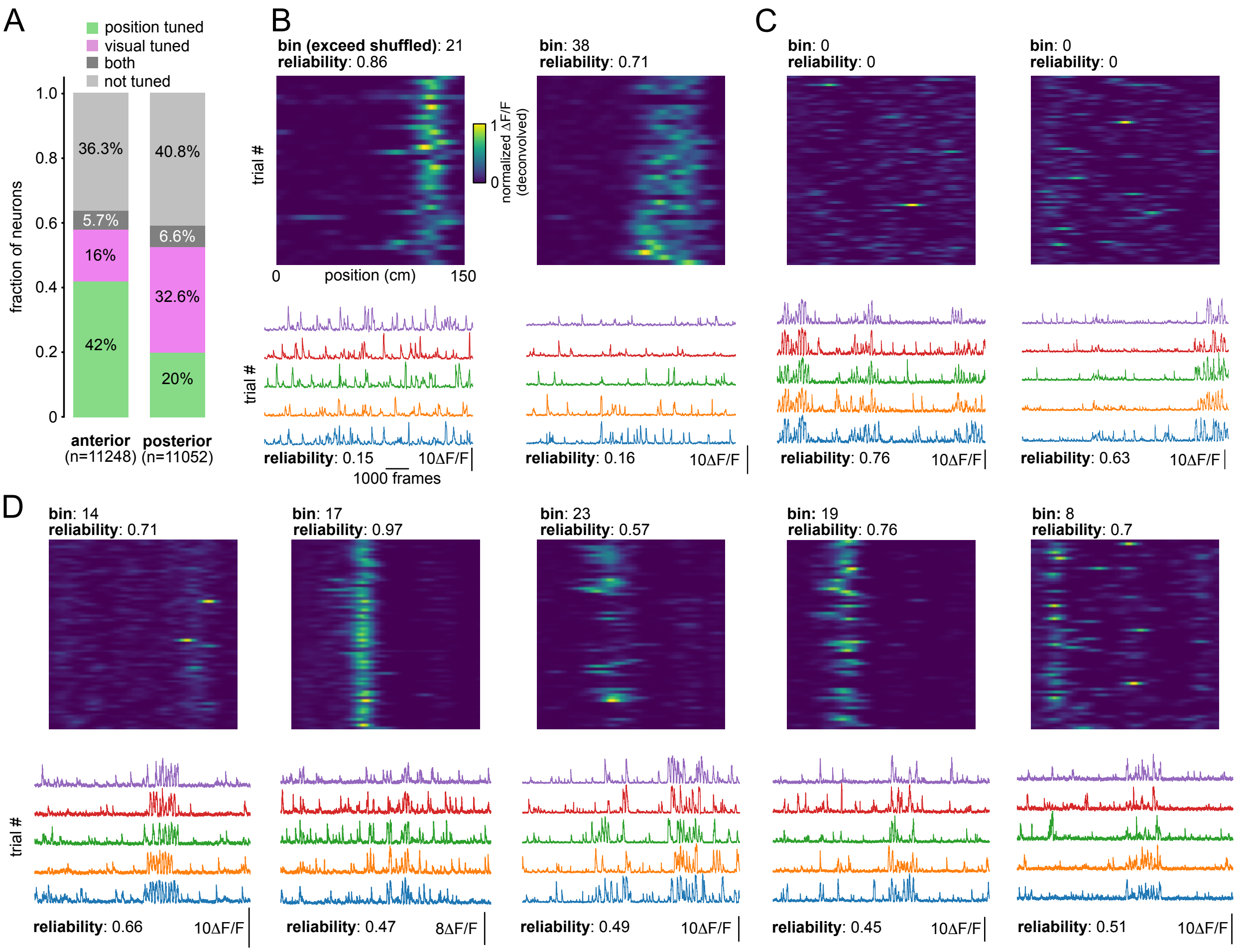

Supplementary Figure 8. Multimodal responses in anterior and posterior RSC

(A) Proportion of neurons showing position-tuned, visually responsive, or bimodal responses in anterior and posterior RSC.

(B–D) Examples of neuron responses: (B) Two position-tuned neurons. (C) Two visually responsive neurons. (D) Five neurons exhibiting both position tuning and visual responsiveness. Heat maps (top) show position-related activity across repeated trials, while the traces show the de-randomized $\Delta F/F_0$ responses to visual stimulation, with different colors representing individual trials.

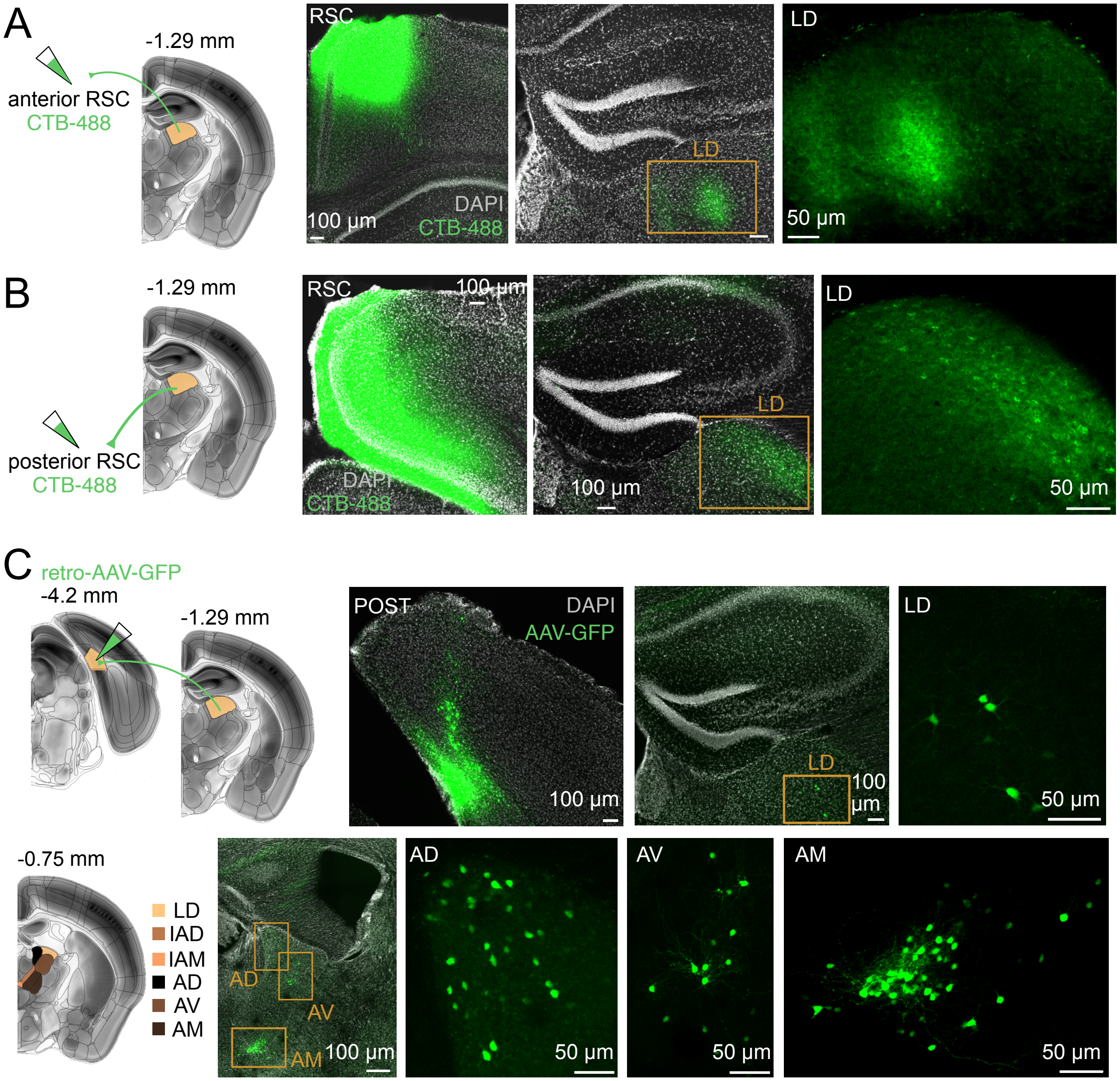

Supplementary Figure 9. Stronger labeling in laterodorsal thalamic input to RSC with CTB compared to AAV

(A) Retrograde labeling in the laterodorsal thalamic nucleus (LD) following injection of CTB-488 into anterior RSC.

(B) Retrograde labeling in LD following injection of CTB-488 into posterior RSC.

(C) Injection site of retrograde AAV (pAAV-CAG-GFP) in the postsubiculum (POST), with corresponding coronal section showing labeled input regions, including the LD thalamus.
