# Supplementary material for: Anterior and posterior retrosplenial cortex form distinct visuospatial circuits in the mouse
Source: Nat Commun. 2026 Mar 25;17:4388. doi: 10.1038/s41467-026-70762-z (PMC13181125; doi:10.1038/s41467-026-70762-z)
Supplement: Supplementary file 2 — Description of Additional Supplementary Files [file 41467_2026_70762_MOESM2_ESM.pdf]

Description of Additional Supplementary Files

Supplementary Movie 1: Widefield calcium imaging showing retinotopic responses in mouse dorsal cortex during presentation of a rotating circular patch stimulus. The video shows fluorescence changes ($\Delta F/F_0$) across the cortical surface as the stimulus moves through different positions in the visual field.
