# Supplementary material for: Anterior and posterior retrosplenial cortex form distinct visuospatial circuits in the mouse
Source: Nat Commun. 2026 Mar 25;17:4388. doi: 10.1038/s41467-026-70762-z (PMC13181125; doi:10.1038/s41467-026-70762-z)
Supplement: Supplementary file 4 — Reporting Summary [file 41467_2026_70762_MOESM4_ESM.pdf]

Corresponding author(s): Vincent Bonin

Last updated by author(s): Jan 19, 2026

Reporting Summary

Nature Portfolio wishes to improve the reproducibility of the work that we publish. This form provides structure for consistency and transparency in reporting. For further information on Nature Portfolio policies, see our Editorial Policies and the Editorial Policy Checklist.

Statistics

For all statistical analyses, confirm that the following items are present in the figure legend, table legend, main text, or Methods section.

n/a	Confirmed	
☐	☒	The exact sample size (*n*) for each experimental group/condition, given as a discrete number and unit of measurement
☐	☒	A statement on whether measurements were taken from distinct samples or whether the same sample was measured repeatedly
☐	☒	The statistical test(s) used AND whether they are one- or two-sided<br>*Only common tests should be described solely by name; describe more complex techniques in the Methods section.*
☐	☒	A description of all covariates tested
☐	☒	A description of any assumptions or corrections, such as tests of normality and adjustment for multiple comparisons
☐	☒	A full description of the statistical parameters including central tendency (e.g. means) or other basic estimates (e.g. regression coefficient) AND variation (e.g. standard deviation) or associated estimates of uncertainty (e.g. confidence intervals)
☐	☒	For null hypothesis testing, the test statistic (e.g. *F*, *t*, *r*) with confidence intervals, effect sizes, degrees of freedom and *P* value noted<br>*Give P values as exact values whenever suitable.*
☐	☒	For Bayesian analysis, information on the choice of priors and Markov chain Monte Carlo settings
☐	☒	For hierarchical and complex designs, identification of the appropriate level for tests and full reporting of outcomes
☐	☒	Estimates of effect sizes (e.g. Cohen's *d*, Pearson's *r*), indicating how they were calculated

Our web collection on statistics for biologists contains articles on many of the points above.

Software and code

Policy information about availability of computer code

Data collection	Scanbox v2 (https://scanbox.org/tag/two-photon-imaging-2/) for calcium imaging; Zen system (Zeiss LSM 900) for confocal scanning
Data analysis	https://github.com/ytsimon2004/rscvp (see the doc: https://rscvp.readthedocs.io/en/latest/; the archived version: https://doi.org/10.5281/zenodo.18234118)

For manuscripts utilizing custom algorithms or software that are central to the research but not yet described in published literature, software must be made available to editors and reviewers. We strongly encourage code deposition in a community repository (e.g. GitHub). See the Nature Portfolio guidelines for submitting code & software for further information.

Data

Policy information about availability of data

All manuscripts must include a data availability statement. This statement should provide the following information, where applicable:

- Accession codes, unique identifiers, or web links for publicly available datasets
- A description of any restrictions on data availability
- For clinical datasets or third party data, please ensure that the statement adheres to our policy

All two-photon imaging datasets (Suite2p-processed) and retrograde-labeling data (CSV format) have been deposited in Zenodo and are publicly available (https://doi.org/10.5281/zenodo.17639283). Any additional data supporting the findings of this study are available from the corresponding author upon request

Research involving human participants, their data, or biological material

Policy information about studies with human participants or human data. See also policy information about sex, gender (identity/presentation), and sexual orientation and race, ethnicity and racism.

Reporting on sex and gender	please see the supplementary table 1
Reporting on race, ethnicity, or other socially relevant groupings	n/a
Population characteristics	n/a
Recruitment	n/a
Ethics oversight	n/a

Note that full information on the approval of the study protocol must also be provided in the manuscript.

Field-specific reporting

Please select the one below that is the best fit for your research. If you are not sure, read the appropriate sections before making your selection.

☒ Life sciences ☐ Behavioural & social sciences ☐ Ecological, evolutionary & environmental sciences

For a reference copy of the document with all sections, see nature.com/documents/nr-reporting-summary-flat.pdf

Life sciences study design

All studies must disclose on these points even when the disclosure is negative.

Sample size	Sample sizes were based on previous studies using similar imaging and tracing methodologies. No statistical methods were used to pre-determine sample sizes. We used 15 animals for functional imaging and 4 animals for anatomical tracing (included supplementary dataset)
Data exclusions	For calcium imaging, as mentioned in the methods, neuron shows poor calcium transients was excluded (see Selection of active neurons). For anatomical tracing, only datasets with clear and robust retrograde expression at the injection site and identifiable labeling in upstream regions were included.
Replication	All key findings were replicated across multiple animals and recording sessions.
Randomization	Randomization was not applicable because all animals underwent the same experimental procedures. Experimental conditions (e.g., imaging region, stimulus type) were systematically varied within subjects to assess regional effects.
Blinding	Blinding was not performed during data collection as experimental conditions were identical across animals. Data analysis was performed using automated pipelines with pre-defined criteria, minimizing potential bias

Reporting for specific materials, systems and methods

We require information from authors about some types of materials, experimental systems and methods used in many studies. Here, indicate whether each material, system or method listed is relevant to your study. If you are not sure if a list item applies to your research, read the appropriate section before selecting a response.

Materials & experimental systems

n/a	Involved in the study	
☒	☐	Antibodies
☒	☐	Eukaryotic cell lines
☒	☐	Palaeontology and archaeology
☐	☒	Animals and other organisms
☒	☐	Clinical data
☒	☐	Dual use research of concern
☒	☐	Plants

Methods

n/a	Involved in the study	
☒	☐	ChIP-seq
☒	☐	Flow cytometry
☒	☐	MRI-based neuroimaging

Animals and other research organisms

Policy information about studies involving animals; ARRIVE guidelines recommended for reporting animal research, and Sex and Gender in Research

Laboratory animals	see supplementary Supplementary Table 1-3 for animal details
Wild animals	n/a
Reporting on sex	See Supplementary Tables 1-3 for detailed information on the animals used. No sex- or gender-based analyses were performed due to the limited number of female mice
Field-collected samples	n/a
Ethics oversight	All animal procedures were approved by the Ethical Committee for Animal Experimentation of KU Leuven and were conducted in accordance with institutional and European guidelines

Note that full information on the approval of the study protocol must also be provided in the manuscript.

Plants

Seed stocks	n/a
Novel plant genotypes	n/a
Authentication	n/a
